# Supplementary figures and images for: Transcriptome analysis of the induction of somatic embryogenesis in Coffea canephora and the participation of ARF and Aux/IAA genes
Source: PeerJ. 2019 Oct 16;7:e7752. doi: 10.7717/peerj.7752 (PMC6800528; doi:10.7717/peerj.7752)

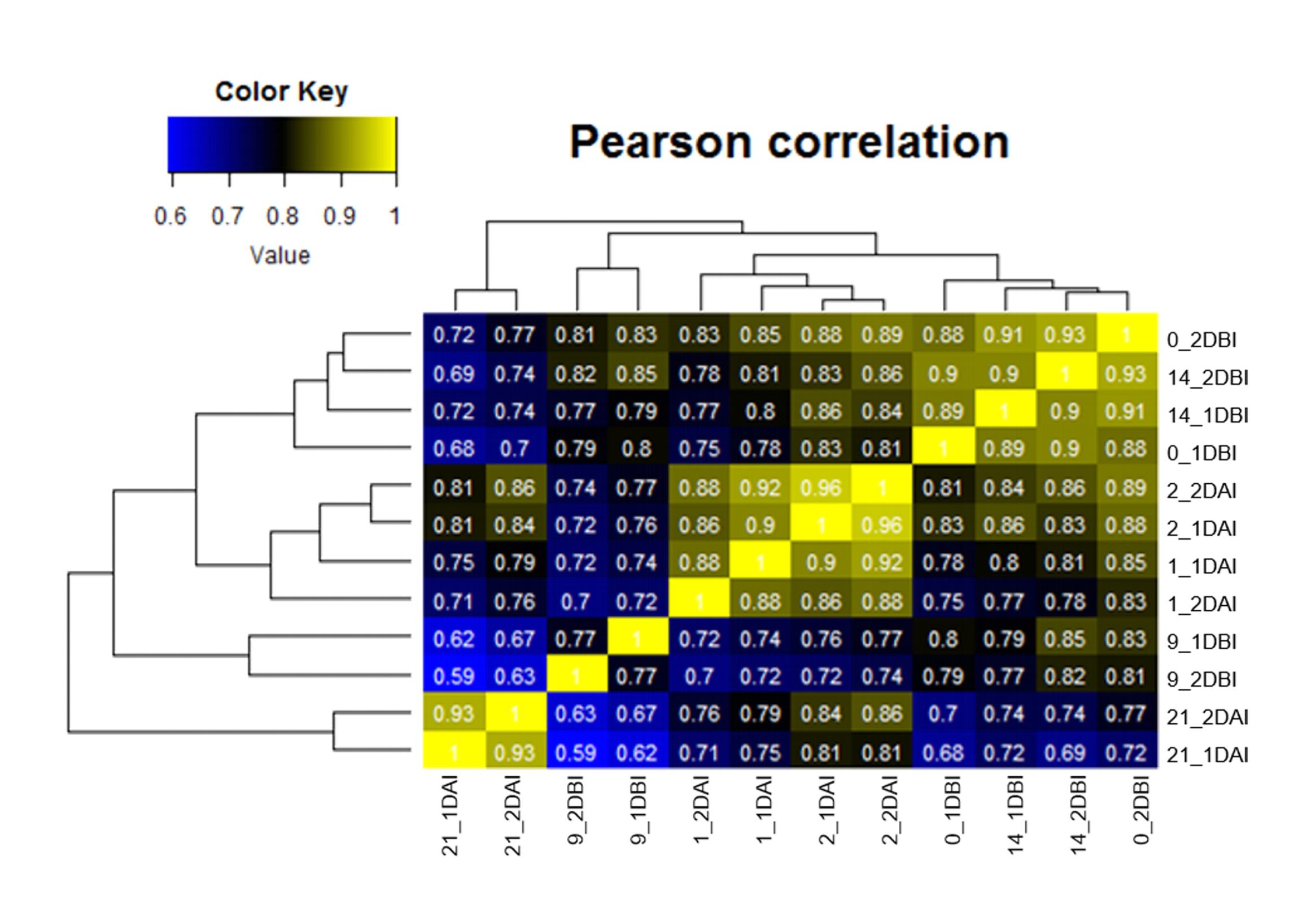

Supplement: Figure S1 [file peerj-07-7752-s001.png]
